# Weight Gain in Infancy and Overweight or Obesity in Childhood across the Gestational Spectrum: a Prospective Birth Cohort Study

**DOI:** 10.1038/srep29867

**Published:** 2016-07-15

**Authors:** Guoying Wang, Sara Johnson, Yiwei Gong, Sarah Polk, Sara Divall, Sally Radovick, Margaret Moon, David Paige, Xiumei Hong, Deanna Caruso, Zhu Chen, Eric Mallow, Sheila O. Walker, Guangyun Mao, Colleen Pearson, Mei-Cheng Wang, Barry Zuckerman, Tina L. Cheng, Xiaobin Wang

**Affiliations:** 1 Department of Population, Family and Reproductive Health, Center on Early Life Origins of Disease, Johns Hopkins University Bloomberg School of Public Health, Baltimore, USA; 2 Division of General Pediatrics & Adolescent Medicine, Department of Pediatrics, Johns Hopkins University School of Medicine, Baltimore, USA; 3 University of Illinois at Urbana-Champaign, Champaign, USA.; 4 Division of Endocrinology, Department of Pediatrics, Johns Hopkins University School of Medicine, Baltimore, USA; 5 School of Environmental Science & Public Health, Wenzhou Medical University Center on Clinical & Epidemiological Eye Research, the Affiliated Eye Hospital of Wenzhou Medical University Wenzhou, China; 6 Department of Pediatrics, Boston University School of Medicine and Boston Medical Center, Boston, USA; 7 Department of Biostatistics, Johns Hopkins University Bloomberg School of Public Health, Baltimore, USA

## Abstract

This study aimed to investigate the optimal degree of weight gain across the gestational spectrum in 1971 children enrolled at birth and followed up to age 7 years. Weight gain in infancy was categorized into four groups based on weight gain *z*-scores: slow (<−0.67), on track (−0.67 to 0.67), rapid (0.67 to 1.28), and extremely rapid (>1.28). Underweight and overweight or obesity (OWO) were defined as a body mass index ≤5^th^ and ≥85^th^ percentile, respectively, for age and gender. In our population, OWO was far more common than underweight (39.7% vs. 3.6%). Weight gain tracked strongly from age 4 to 24 months, and was positively associated with OWO and an unfavorable pattern of metabolic biomarkers, although the degree of weight gain for the risk was different across gestational categories. Extremely rapid weight gain led to a particularly high risk of OWO among children born early term and late preterm: odds ratio: 3.3 (95% confidence interval: 1.9 to 5.5) and 3.7 (1.8 to 7.5), respectively, as compared to those with on track weight gain. Our findings suggest that monitoring and ensuring optimal weight gain across the entire gestational spectrum beginning from birth represents a first step towards primary prevention of childhood obesity.

The persistently high prevalence of obesity is a major clinical and public health challenge in the U.S. and globally. Of concern, 8.1% of U.S. infants and toddlers were overweight, and 16.9% of 2- to 19-year-olds were obese in 2011–2012^1^. More importantly, obesity in young children leads not only to short-term morbidity but also to later obesity and its adverse consequences across the lifespan and generations^2^,^3^,^4^,^5^. Growing evidence indicates that obesity may originate in early life. A recent study has lent even further evidence, showing that incident obesity between age 5 and 14 years was more likely to originate at younger ages^6^. However, questions remain regarding what modifiable early life factors can increase the risk of childhood obesity.

Growth in early infancy is more rapid than at any other time during postnatal life. In particular, infants born preterm or early term usually compensate by engaging in rapid “catch-up” growth in the first year of life. Although studies of term births suggest that rapid weight gain in early life (ranging from 6 to 24 months) is associated with an increased risk of childhood obesity^7^,^8^, there is a lack of prospective birth cohort studies to investigate whether the associations persist across the entire gestational spectrum. Pediatricians monitor infant weight gain closely for “failure to thrive” and make recommendations to increase calories in babies born preterm. However, most current recommendations regarding appropriate growth velocity in infancy and early childhood largely do not consider the long term risk of obesity or metabolic disorders, nor do these recommendations address an infants’ specific gestational age category^9^. Moreover, current recommendations regarding the age to begin screening for obesity in children do not cover infancy: the U.S. Preventive Services Task Force recommends screening by age 6 years^10^, whereas the Expert Committee recommends screening by age 2 years^11^.

Using a prospective birth cohort enriched by preterm births, we aimed to investigate weight gain patterns and their associations with the risk of both underweight and OWO as well as metabolic biomarkers during early childhood (median [Interquartile range] age: 6[4–7] years) among children born across the gestational spectrum (full term, early term, late preterm and early preterm). This line of investigation is needed to provide evidence to establish optimal growth targets to balance the need for normal or catch-up growth and reduce the risk of OWO in children. Such information is critical to help identify children at high-risk of developing OWO during early infancy when interventions could be highly cost-effective and have lifelong impact.

## Results

### Characteristics of the study participants

Of the 1971 children (980 boys, 991 girls), 1442 were born term (926 full term, 516 early term) and 529 were born preterm (300 late preterm, 229 early preterm). Prenatal and postnatal anthropometric and growth parameters are presented in Table 1. Compared to those born full term, infants born preterm had lower birthweights and their mothers had higher rates of smoking, diabetes and hypertensive disorders during pregnancy. In total, 39.7% of the children (39.2% of boys and 40.2% of girls) were OWO, whereas only 72 (3.6%) children were underweight at age 2–7 years.

### Persistence of weight gain during infancy

**Figure 1:**
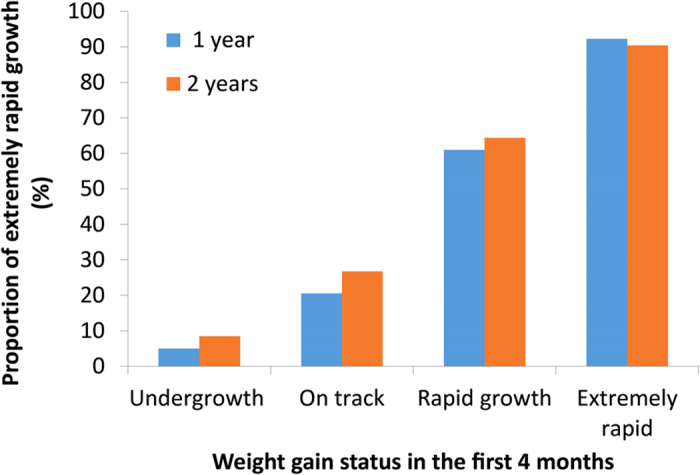
Proportion of extremely rapid weight gain in the first year and first 2 years according to weight gain status in the first 4 months of life. Weight gain *z*-score was defined as the change in weight for age *z*–score between birth and age 4 months and was categorized into four groups: slow (weight gain *z*-score less than −0.67); on track (weight gain *z*-score between −0.67 and 0.67), rapid (weight gain *z*-score between 0.67 and 1.28), and extremely rapid (weight gain *z*-score greater than 1.28).

Infants born preterm had a higher percentage of rapid or extremely rapid weight gain in the first four months of life relative to babies born at term (76.4% vs. 29.5%); the highest rate was among early preterm births (87.8%). Data from all three time points (first 4 mo, 1y, and 2y) were available for 1643 study participants. Weight gain was highly correlated for the first four months and the first year (r = 0.81, p < 0.001), as well as for the first four months and at age 2 years (r = 0.74, p < 0.001). Rapid and extremely rapid weight gain tracked well across all of infancy. Rapid or extremely rapid weight gain persisted from the first four months to age 2 years (Fig. 1). In total, 94.5% and 91.5% of children who experienced rapid or extremely rapid growth in the first four months remained on the same track at years 1 and 2, respectively.

### Weight gain, Body Mass Index (BMI) *z*-score and the risk of overweight or obesity

**Figure 2:**
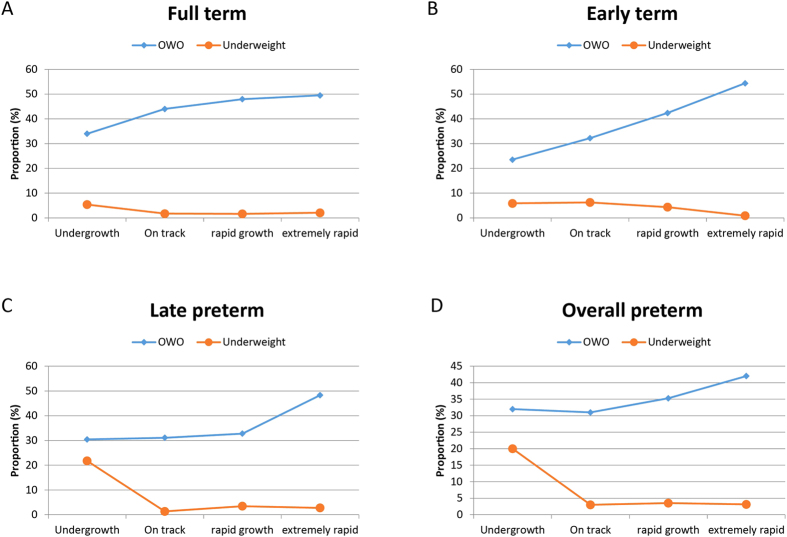
The relationship between weight gain z-score in the first 4 months of life and the percentage of underweight and overweight or obesity at age 2–7 years stratified by gestational age category. Gestational age category was defined as full term (gestational age ≥39 weeks), early term (37–38 weeks), late preterm (34–36 weeks), and early preterm (<34 weeks). Full term (panel A); early term (panel B); late preterm (panel C); overall preterm (panel D).

Weight gain *z*-score in the first four months of life was positively associated with BMI *z*-score and OWO at age 2–7 years. The overall risk of OWO increased by 50% for every increase of one standard deviation (SD) in weight gain in the first four months (multivariate odds ratio [OR], 1.5; 95% confidence interval [CI], 1.4 to 1.7). The corresponding ORs (95%CI) were 1.9 (1.6 to 2.3) and 1.8 (1.4 to 2.3) for children who were born early term (37–38 weeks) and late preterm birth (34–36 weeks), respectively. When weight gain in the first four months of life was further categorized into four subgroups (on track, slow, rapid, and extremely rapid), extremely rapid weight gain was associated with a 2.3 (95%CI, 1.7 to 3.0) times greater risk of OWO than the on track group after adjusting for prenatal and postnatal risk factors (maternal race/ethnicity, educational attainment, smoking, parity, prepregnancy BMI category, diabetes status, hypertensive disorder, fetal growth status, gestational age categories, and breastfeeding) (Table 2). For full term infants, extremely rapid weight gain increased the risk of OWO by 60%, OR = 1.6, 95%CI, 1.0 to 2.4. An increased risk of OWO associated with extremely rapid weight gain appeared to be especially prominent among participants who were born early term and late preterm. The corresponding ORs (95%CI) were 3.3 (1.9 to 5.5) and 3.7 (1.8 to 7.5) for children born early term and late preterm, respectively (Table 2). Notably, the association pattern showed a difference across the gestational spectrum. In those born full term, children with both rapid weight gain and extremely rapid growth were at a similarly high risk of OWO. In those born early term, extremely rapid growth further increased the risk of OWO beyond those with rapid growth. However, in those born late preterm, rapid growth was not associated with OWO, but extremely rapid growth significantly increased the risk of OWO. In contrast, in those born early preterm, even extremely rapid weight gain was not associated with the risk of OWO (Fig. 2). Of note, after additional adjustment for duration of breastfeeding and timing of solid food introduction, the results did not substantially change. At the same time, excessive weight gain showed no clear benefit for reducing underweight, even among preterm or early term infants who need catch-up growth (Fig. 2).

### Sensitivity analysis

When we repeated our primary analyses stratified by child’s age group (2–4 years and 5–7 years) the findings were substantially unchanged (Table 3). Likewise, when we examined the associations between weight gain in the first and second year relative to the risk of OWO, the associations were consistent. Point estimates were slightly higher when 1-year or 2-year windows of weight gain were used in the analyses rather than the shorter 4-month window (Table 4).

### Weight gain and metabolic biomarkers: plasma leptin, adiponectin and adiponectin/leptin ratio

In sum, 1136 participants were available for the analyses of blood leptin concentrations, 1139 for adiponectin, and 1092 for the adiponectin/leptin ratio. The median (Interquartile range) age of biomarker measurement was 1.7 (0.9–3.5) years. Weight gain *z*-score in the first four months was positively associated with plasma leptin, whereas it was negatively associated with the adiponectin/leptin ratio. Every one unit increase in the weight gain z-score was associated with a 0.18 unit (log-transformed) increase in plasma leptin, and a 0.17 unit decrease in the adiponectin/leptin ratio (log-transformed). The associations were similar across the entire gestational spectrum. A corresponding 0.19 and 0.23 unit increase in leptin and a 0.22 and 0.27 unit decrease in the adiponectin/leptin ratio were observed among early term and late preterm children, respectively (Table 5).

## Discussion

In the U.S., preterm birth affects 1 in 9 of all live births and 1 in 5 Black infants^12^. To date, the majority of studies that have associated rapid weight gain in early life with later obesity have been performed in children and adults born at term^7^,^13^,^14^. Although those born preterm display a faster rate of infant growth, data regarding the linkage between rapid weight gain in early life and the risk of childhood and adult obesity among preterm births are limited. Recently, the American Congress of Obstetricians and Gynecologists (ACOG) recommended defining children who are born between at 37–38 weeks as early term births; this group of births has received increased attention due to their increased risk of morbidity in the early and later postnatal period^15^.

To our knowledge, this is the first large-scale birth cohort study to investigate the effect of rapid weight gain in infancy on the risk of childhood OWO, as well as on metabolic biomarkers in children born preterm and early term, in a U.S., predominantly urban minority population. Our analyses showed a high prevalence of childhood OWO in this racial/ethnic minority population, which was 1.7 times higher than the rates for U.S. children aged 2–5 years^1^. The higher prevalence of OWO in our sample is consistent with the well documented socioeconomic and racial/ethnic disparities for childhood obesity^16^. The prevalence of underweight was much lower in our study population, especially among term births. Our data also showed a remarkable tracking of rapid or extremely rapid growth in infancy across the first two years of life, suggesting that monitoring and ensuring optimal weight gain should begin at birth.

We found that rapid weight gain during infancy was associated with an increased risk of OWO at age 2–7 years in a dose-response fashion among all children except for early preterm births, even after adjusting for pertinent prenatal and postnatal covariables. Most remarkably, we found that the effect of excessive weight gain on childhood OWO appeared to be more pronounced in children born early term or late preterm. However, excessive weight gain showed no clear benefit for reducing underweight, even among preterm or early term infants who need catch-up growth. In light of this finding, we should be sure not to lose sight of the potential adverse effects of inadequate weight gain, particularly on neurodevelopmental outcomes^17^. Given that a low degree of rapid weight gain did not increase the risk of OWO in those born late preterm, optimal catch-up growth for those infants is recommended. More importantly, since rapid weight gain may benefit neurodevelopment and did not increase the risk of OWO in early preterm births, catch-up growth is seen to be critical for early preterm infants. Our findings underscore the importance of identifying an “optimal” weight gain to help establish a balance between the need to prevent childhood obesity and metabolic consequences versus the need to ensure healthy neurodevelopment.

Adipose tissue has been recognized as an important endocrine organ that secretes adipokines. Two major adipokines, leptin and adiponectin, are thought to play important roles in the regulation of insulin sensitivity and metabolic homeostasis. Previous studies have shown that plasma leptin concentrations increase with obesity, whereas plasma adiponectin concentrations are decreased in obese individuals^18^,^19^. The leptin/adiponectin ratio is strongly correlated with the glucose infusion rate as measured by the euglycemic hyperinsulinemic clamp method^20^. In children, a high leptin/adiponectin ratio is associated with cardiovascular risk and systematic inflammation^21^. More importantly, studies have also demonstrated that the leptin/adiponectin ratio serves as a marker of insulin resistance that is superior to leptin or adiponectin alone^20^,^22^,^23^. Conversely, the adiponectin/leptin ratio serves as a marker of insulin sensitivity^24^. It is well understood that BMI is not an exact measure of adiposity, especially in young children^25^,^26^. Although it is conventionally accepted, the accuracy of the diagnosis of obesity based on BMI categories is limited. Therefore, we determined obesity-related biomarkers to further verify our findings. We measured plasma leptin concentrations (as a surrogate of body fat) and adiponectin concentrations (as a marker of insulin sensitivity). We also calculated the adiponectin/leptin ratio as an alternative marker of insulin sensitivity. Our data showed that weight gain in the first four months was significantly associated with increased plasma leptin levels and a decreased adiponectin/leptin ratio, but the associations with plasma adiponectin concentrations alone were weak. These findings suggest that the adiponectin/leptin ratio, which indicates the relative abundance of adiponectin over leptin, may present the best way to capture their combined contribution to the association with child weight gain.

Our findings have important clinical and public health implications. Adequate weight gain in preterm infants appears to benefit neurodevelopment^27^. However, despite the potential benefit of “catch-up” growth, our study revealed that extremely rapid weight gain in the first four months of life is a risk factor for OWO in all childhood except those born early preterm, and particularly for those born early term and late preterm. Our findings underscore the premise that optimal weight gain targets in early infancy may benefit from being tailored to a specific gestational age and to first 4-month weight gain trajectories. These efforts may help to reduce childhood obesity and minimize adverse metabolic consequences in later life.

The latest American Academy of Pediatrics (AAP) report emphasizes the critical role of pediatricians in preventing childhood obesity^28^. Between infancy and a baby’s first birthday, there are a total of seven recommended well-child visits. However, the recommendations regarding the age to begin screening for obesity in children do not currently cover infancy: the U.S. Preventive Services Task Force recommends screening by age 6 years^10^ whereas the Expert Committee recommends screening much earlier—by age 2 years^11^. However, numerous studies including our own have recognized the prenatal and early childhood stages as being sensitive periods in the development of obesity because of the rapid cellular proliferation and differentiation and metabolic programming that occurs during this time^29^,^30^. In addition, several studies have documented that the proliferation and differentiation of adipocytes occurs primarily *in utero*^31^, and that the total number of adipocytes, a major determinant of adult fat mass, is established in early childhood^32^,^33^,^34^. Previous studies also have shown an increased risk of obesity in older children following rapid weight gain as early as the first 4–6 months of life in term births^7^,^13^,^14^, and incident obesity between the ages of 5 and 14 years subsequent to being overweight in kindergarten^6^. Our study findings raise the prospect that the first steps toward primary prevention of OWO might need to occur very early in infancy, and that conducting rigorous studies of early life risk factors and intervention targets would not only be an important step in reducing OWO but could yield commensurate long-term health benefits.

Some particular strengths of this study were that it was based on a large prospective birth cohort. The fact that the study sample was enriched with preterm births enabled us to examine weight gain patterns in infancy among infants born early term and preterm. This study was further strengthened by the measurements of metabolic biomarkers, leptin and adiponectin. Our study also had some limitations. The year of participant enrollment varied due to rolling recruitment in the Boston Birth Cohort. However, we examined growth parameters based on gender- and age-specific *z*-scores, which allowed us to account for variation in age at follow-up. Because we did not quantify the intake of caloric or specific macro- or micronutrients during infancy, we could not examine the effects of these on weight gain patterns and the risk of childhood obesity.

## Conclusions

We demonstrated a high prevalence of OWO across the gestational age spectrum within this urban, predominantly low-income minority sample. The association patterns of weight gain in infancy with childhood OWO were different according to gestational age categories. Extremely rapid weight gain in the first four months of life was a strong predictor of OWO at age 2–7 years for all children except early preterm births. Because early infancy is a critical period in which to establish an optimal growth trajectory and prevent childhood obesity, as we observed in this study, an optimal growth pattern should be established according to gestational age. Pediatricians can play an important role in monitoring and educating families to achieve optimal weight gain in infants beginning at birth.

## Methods

### Study population

From 2004–2014, we followed 2937 children who were born at the Boston Medical Center, MA, between 1998–2012 and recruited into the Boston Birth Cohort, a large, prospective, predominantly urban minority birth cohort^35^. We excluded 740 participants due to missing a visit in the first four months and 226 participants due to missing a visit during years 2–7. The final study analyses included 1971 (67% of the total follow-up children) participants who had a visit within the first four months of life and at least one follow-up visit from age 2–7 years. Children who were included relative to the total follow-up sample in the study were similar with regard to prenatal variables as well as birth outcomes. The evaluation of enrollment and follow-up included a medical questionnaire and interview and anthropometric measurements. The study protocol was approved by the Institutional Review Boards of the Boston University Medical Center, the Ann & Robert H. Lurie Children’s Hospital of Chicago (formerly Children’s Memorial Hospital), and the Johns Hopkins University Bloomberg School of Public Health. The methods were carried out in accordance with the approved guidelines. Written informed consent was obtained from mothers.

### Measurement of major variables and outcomes

#### Major Prenatal and Postnatal Variables

Maternal race/ethnicity, educational attainment, smoking, parity and prepregnancy weight and height were collected via structured interview 24 to 72 hours after delivery. Maternal prepregnancy BMI was calculated as weight in kilograms divided by height in meters squared. Maternal smoking during pregnancy was coded as “never a smoker”, “quit for the pregnancy”, or “continuous smoker” based on whether the mother smoked cigarettes throughout the pregnancy, only smoked in the three months before pregnancy, or only smoked during the first trimester^35^. Race/ethnicity was based on maternal response to fixed categories in the questionnaire and classified as Black, Hispanic, or other. Parity was grouped into nulliparous and multiparous. Maternal diabetes status was categorized as either “pre-gestational/gestational diabetes” or “no diabetes” based on the medical record^36^. Hypertensive disorders were coded as being present if the mother had experienced one or more of the following: pre-eclampsia, eclampsia, chronic hypertension or hemolysis, elevated liver enzymes, or low platelet (HELLP) syndrome^37^. Information on infant feeding was obtained using a standardized interview performed at follow-up study visits over the first few years of life, and subjects were classified into three groups: (1) formula-fed exclusively, (2) breast-fed exclusively, or (3) both^38^.

#### Gestational Age Category

Gestational age was assessed based on the last menstrual period and verified by early ultrasound (<20 weeks of gestation)^35^. Term births were defined as gestational age ≥37 weeks, and were further categorized into two groups: full term (gestational age ≥39 weeks) and early term (37–38 weeks). Preterm births were defined as gestational age <37 weeks, and were further grouped into late preterm (34–36 weeks) and early preterm (<34 weeks).

#### Measures of Infancy Weight Gain and Overweight or Obesity

Child height and weight were obtained at well-child visits as part of pediatric primary care at the Boston Medical Center. Weight-for-age *z*-scores in the first two years were calculated using WHO reference values^39^. Weight gain *z*-scores in infancy were defined as the change in weight-for-age *z*-scores from birth to the target time points, and were categorized into four groups: slow (weight gain *z*-score <−0.67), on track (−0.67 to 0.67), rapid (>0.67 to 1.28), and extremely rapid (>1.28). These groups are equivalent to “crossing downward one or more”, “no crossing”, “crossing upwards one” and “crossing upwards two or more” of the major weight percentile lines (2^rd^, 10^th^, 25^th^, 50^th^, 75^th^, 90^th^, and 98^th^) on the WHO standard growth chart, respectively.

BMI *z*-scores and percentiles were calculated using U.S. national reference data^39^. Although there are different growth charts for term and preterm infants, we chose one national standard for all study participants because our objective was to assess the association between weight gain in early infancy and childhood obesity across the gestational age spectrum. Underweight and OWO were defined as BMI <5^th^ and BMI ≥85^th^ percentile, respectively, for age and gender based on the U.S. Centers for Disease Control and Prevention (CDC) definition^40^.

#### Leptin and Adiponectin Measures

Leptin concentrations were determined using sandwich immunoassays based on flow metric xMAP technology on Luminex 200 machines (Luminex Corp., Austin, TX) with an inter-assay coefficient of variation (CV) of 4.5%. Adiponectin was measured by ELISA with an inter-assay CV of <5.8%. The immunoassay kit was obtained commercially from Millipore Corp. Each sample was run in duplicate, and the intra-assay CVs for leptin and adiponectin were 4.3% and 2.9%, respectively.

### Statistical analysis

We examined participant characteristics and outcomes by gestational age category. To calculate unadjusted trend *P* values across gestational age category, we used the Mantel-Haenszel χ^2^ test for categorical characteristics and linear regression for continuous characteristics. Weight gain *z*-score was evaluated both as a continuous variable and as a categorical variable by weight gain category (slow, on track, rapid and extremely rapid weight gain). Pearson correlation coefficients were computed to examine the association between weight gain *z*-score from age 4 to 24 months. In order to examine the independent effects of weight gain on BMI *z*-scores, multiple linear regressions were applied to the total samples and four subgroups (full term, early term, late preterm, and early preterm) and adjusted for prenatal and postnatal risk factors including maternal race/ethnicity, educational attainment, smoking status, parity, pre-pregnancy BMI, pre-gestational or gestational diabetes, hypertensive disorders, fetal growth status, and infant breastfeeding status. For total sample models, we additionally adjusted for gestational age categories. Similarly, multiple logistic regressions were used to examine the effects of weight gain on the risk of OWO, adjusted for the aforementioned covariables. As the weight gain *z*-scores, BMI *z*-scores and BMI percentiles already controlled for child age and sex, analyses were not further adjusted for these parameters.

We performed several sensitivity analyses to test for the influence of child age on the above associations by stratifying the age group (2–4 years, 5–7 years). In order to examine the impact of the time window of infancy weight gain, we expanded the time window for calculating the average weight gain levels (to 1 or 2 years rather than 4 months). Those associations with *P* values (2-sided tests) less than 0.05 were regarded as statistically significant. All analyses were conducted using SAS version 9.3 (SAS Institute, Cary, NC).

## Additional Information

**How to cite this article**: Wang, G. *et al*. Weight Gain in Infancy and Overweight or Obesity in Childhood across the Gestational Spectrum: a Prospective Birth Cohort Study. *Sci. Rep.* **6**, 29867; doi: 10.1038/srep29867 (2016).

## Figures and Tables

**Table 1:** The characteristics of the study population.

	**Term**		**Preterm**		**P for trend**
	**Full tem (>39 wk)**	**Early term (37–38 wk)**	**Late preterm (34–36 wk)**	**Early preterm (<34 wk)**	
No.	926	516	300	229	
Maternal characteristics					
Age at enrollment, y	28.2 ± 6.4	29.0 ± 6.9	29.0 ± 6.5	28.9 ± 6.5	0.029
Race, No. (%)					0.015
Black	575(62.1)	328(63.7)	194(64.7)	159(69.4)	
Hispanic	170(18.4)	88(17.1)	56(18.7)	45(19.7)	
Other	181(19.5)	99(19.2)	50(16.6)	25(10.9)	
Education, No. (%)					0.449
High school or less	619(66.9)	311(60.3)	213(71.0)	140(61.1)	
College or higher	307(33.1)	205(39.7)	87(29.0)	89(38.9)	
Smoking, No. (%)					<0.001
Never	785(84.8)	430(83.4)	225(75.0)	179(78.2)	
Quitter	68(7.3)	27(5.2)	28(9.3)	24(10.5)	
Continuous	73(7.9)	59(11.4)	47(15.7)	26(11.3)	
Parity					0.737
Nulliparous	405(43.7)	204(39.5)	130(43.3)	98(42.8)	
Multiparous	521(56.3)	312(60.5)	170(56.7)	131(57.2)	
Prepregnancy BMI category, No. (%)					0.235
<25 kg/m2	433(46.8)	259(50.2)	144(48.0)	97(38.0)	
25–29.9 kg/m2	263(28.4)	146(28.3)	81(27.0)	86(37.5)	
≥30 kg/m2	230(24.8)	111(21.5)	75(25.0)	56(24.5)	
Diabetes status, No. (%)	85(9.2)	72(14.2)	45(15.0)	31(13.5)	0.05
Hypertensive disorders, No. (%)	80(8.6)	93(18.0)	75(25.0)	84(36.7)	<0.001
Child characteristics					
Gender, No. (%)					0.447
Male	450(48.6)	257(49.8)	162(54.0)	111(48.5)	
Female	476(51.4)	259(50.2)	138(46.0)	118(51.5)	
Gestational age, wk	40.1 ± 0.8	38.1 ± 0.6	35.7 ± 0.9	29.8 ± 3.0	<0.001
Birthweight, g	3395 ± 492	2966 ± 514	2502 ± 552	1400 ± 569	<0.001
Birthweight for gestational age category, No. (%)					0.013
Small for gestational age	108(11.6)	92(17.8)	40(13.3)	9(3.9)	
Appropriate for gestational age	719(77.7)	382(74.0)	229(76.3)	205(89.5)	
Large for gestational age	99(10.7)	42(8.1)	31(10.3)	15(6.6)	
Breastfed, No. (%)					0.835
Formula	221(23.9)	119(23.0)	82(27.4)	60(26.2)	
Breast-fed	61(6.6)	31(6.0)	16(5.3)	7(3.1)	
Both	637(68.8)	362(70.2)	201(67.0)	159(69.4)	
Missing	7(0.7)	4(0.8)	1(0.3)	3(1.3)	
Rapid or extremely rapid weight gain, No. (%)					
In the first 4 mo	220(23.8)	206(39.9)	203(67.7)	201(87.8)	<0.001
In the first 1 y^*^	338(38.5)	291(60.1)	241 (86.4)	215(98.6)	<0.001
In the first 2 y^†^	333(41.5)	277(61.8)	229(87.7)	201(100.0)	<0.001
OWO, No. (%)	388(41.9)	192(37.2)	119(39.7)	84(36.7)	0.129
Underweight, No. (%)	27(2.9)	24(4.6)	12(4.0)	9(3.9)	0.291

Data are presented as mean ± SD or No. (%); BMI, body mass index; OWO, overweight or obesity; Hypertensive disorder was defined as being present if the mother had experienced one or more of the following: pre-eclampsia, eclampsia, chronic hypertension or hemolysis, elevated liver enzymes, or low platelet (HELLP) syndrome. ^*^112 values missing; ^†^258 values missing.

**Table 2:** The association between weight gain in the first 4 months and BMI *z*-score and overweight or obesity at age 2–7 years.

**Weight gain category**	**N**	**BMI z-score**		**Overweight or obesity**	
		**mean** ± **SD**	**β(95%CI)**	**Case, No. (%)**	**OR(95%CI)**
Total sample*					
On track	717	0.62 ± 1.20	ref	278(38.8)	1.0
Slow growth	424	0.45 ± 1.30	−0.34(−0.48, −0.20)^§^	133(31.4)	0.5(0.4, 0.7)^§^
Rapid growth	300	0.78 ± 1.20	0.36(0.20, 0.52)^§^	128(42.7)	1.7(1.2, 2.2)^§^
Extremely rapid	530	0.89 ± 1.24	0.57(0.42, 0.72)^§^	244(46.0)	2.3(1.7, 3.0)^§^
Weight gain *z*-score			0.26(0.22, 0.30)^§^		1.5(1.4, 1.7)^§^
Full term					
On track	409	0.76 ± 1.17	ref	180 (44.0)	1.0
Slow growth	297	0.53 ± 1.26	−0.32(−0.49, −0.16)^§^	101(34.0)	0.5(0.4, 0.8)^‡^
Rapid growth	123	0.97 ± 1.10	0.37(0.15, 0.59)^‡^	59(48.0)	1.5(1.0, 2.4)
Extremely rapid	97	1.04 ± 1.18	0.39(0.14, 0.64)^‡^	48(49.5)	1.6(1.0, 2.6)
Weight gain *z*-score			0.21(0.15, 0.27)^§^		1.4(1.2, 1.6)^§^
Early term					
On track	208	0.45 ± 1.19	ref	67(32.2)	1.0
Slow growth	102	0.32 ± 1.33	−0.33(−0.61, −0.05)^†^	24(23.5)	0.4(0.2, 0.8)^‡^
Rapid growth	92	0.72 ± 1.27	0.34(0.06, 0.62) ^†^	39(42.4)	1.7(1.0, 3.0)
Extremely rapid	114	1.06 ± 1.15	0.71(0.45, 0.98) ^§^	62(54.4)	3.2(1.9, 5.5)^§^
Weight gain *z*-score			0.31(0.22, 0.39)^§^		1.9(1.6, 2.3)^§^
Late preterm					
On track	74	0.48 ± 1.23	ref	23(31.1)	1.0
Slow growth	23	0.01 ± 1.65	−0.71(−1.26, −0.16)^†^	7(30.4)	0.5(0.1, 1.8)
Rapid growth	58	0.57 ± 1.27	0.27(−0.14, 0.68)	19(32.8)	1.5(0.6, 3.5)
Extremely rapid	145	0.95 ± 1.23	0.70(0.36, 1.04)^§^	70(48.3)	3.6(1.8, 7.5)^§^
Weight gain *z*-score			0.35(0.25, 0.46)^§^		1.8(1.4, 2.3)^§^
Early preterm					
On track	26	0.25 ± 1.53	ref	8(30.8)	1.0
Slow growth	2	0.49 ± 1.28	–	1(50.0)	–
Rapid growth	27	0.53 ± 1.11	0.49(−0.21, 1.19)	11(40.7)	2.2(0.6, 7.8)
Extremely rapid	174	0.65 ± 1.32	0.49(−0.05, 1.03)	64(36.8)	1.5(0.5, 4.0)
Weight gain *z*-score			0.27(0.13, 0.42)^§^		1.3(1.0, 1.7)

Gestational age category was defined as full term (gestational age ≥39 weeks), early term (37–38 weeks), late preterm (34–36 weeks), and early preterm (<34 weeks). Weight gain *z*-score was defined as the change in weight for age *z*–score between birth and 4 months and was categorized into four groups: slow (weight gain *z*-score less than −0.67); on track (weight gain *z*-score between −0.67 and 0.67), rapid (weight gain *z*-score between 0.67 and 1.28), and extremely rapid (weight gain *z*-score more than 1.28).

Adjusted for maternal education, race, smoking, parity, prepregnancy BMI category, hypertensive disorders, diabetes, fetal growth status, and breastfeeding status.

*Additional adjustment for gestational age categories. ^†^P < 0.05; ^‡^P < 0.01; ^§^P < 0.001.

**Table 3:** The association between weight gain in the first 4 months and BMI *z*-score and overweight or obesity at age 2–4 years and 5–7 years.

**Weight gain category**	**N**	**BMI z-score**		**Overweight or obesity**	
		**mean** ± **SD**	**β(95%CI)**	**Case, No. (%)**	**OR(95%CI)**
Age 2–4 years					
On track	706	0.51 ± 1.33	ref	237(33.6)	1.0
Slow growth	412	0.36 ± 1.38	−0.35(−0.50, −0.20)^§^	128(31.1)	0.7(0.5, 0.9)^‡^
Rapid growth	294	0.69 ± 1.26	0.43(0.26, 0.61)^§^	112(38.1)	1.8(1.3, 2.4)^§^
Extremely rapid	516	0.86 ± 1.34	0.76(0.60, 0.92)^§^	239(46.3)	3.3(2.5, 4.4)^§^
Weight gain *z*-score			0.32(0.28, 0.37)^§^		1.6(1.5, 1.8)^§^
Age 5–7 years					
On track	468	0.68 ± 1.20	ref	199(42.5)	1.0
Slow growth	283	0.60 ± 1.26	−0.20(−0.37, −0.04)^†^	102(36.0)	0.6(0.4, 0.9)^‡^
Rapid growth	192	0.94 ± 1.11	0.46(0.27, 0.65)^§^	91(47.4)	1.7(1.2, 2.4)^‡^
Extremely rapid	336	1.05 ± 1.18	0.62(0.44, 0.79)^§^	171(50.9)	2.1(1.5, 3.0)^§^
Weight gain *z*-score			0.24(0.19, 0.29)^§^		1.5(1.3, 1.6)^§^

Gestational age category was defined as full term (gestational age ≥39 weeks), early term (37–38 weeks), late preterm (34–36 weeks), and early preterm (<34 weeks). Weight gain *z*-score was defined as the change in weight for age *z*–score between birth and 4 months and was categorized into four groups: slow (weight gain *z*-score less than −0.67); on track (weight gain *z*-score between −0.67 and 0.67), rapid (weight gain *z*-score between 0.67 and 1.28), and extremely rapid (weight gain *z*-score more than 1.28).

Adjusted for maternal education, race, smoking, parity, prepregnancy BMI category, hypertensive disorders, diabetes, fetal growth status, gestational age categories, and breastfeeding status.

^†^P < 0.05; ^‡^P < 0.01; ^§^P < 0.001.

**Table 4:** The association between weight gain in the first year and first 2 years and BMI *z*-score and overweight or obesity at age 2–7 years.

**Weight gain category**	**N**	**BMI z-score**		**Overweight or obesity**	
		**mean ± SD**	**β(95%CI)**	**Case, No. (%)**	**OR(95%CI)**
Weight gain in first year category					
On track	577	0.52 ± 1.22	ref	196(34.0)	1.0
Slow growth	197	0.35 ± 1.22	−0.45(−0.63, −0.26)^§^	56(28.4)	0.5(0.3, 0.7)^§^
Rapid growth	286	0.66 ± 1.20	0.31(0.15, 0.48)^§^	116(40.6)	1.8(1.3, 2.5)^§^
Extremely rapid	799	0.88 ± 1.24	0.78(0.63, 0.92)^§^	372(46.6)	3.7(2.8, 4.9)^§^
Weight gain *z*-score			0.33(0.28, 0.37)^§^		1.7(1.6, 1.9)^§^
Weight gain in first 2 years category					
On track	508	0.53 ± 1.20	ref	173(34.1)	1.0
Slow growth	165	0.15 ± 1.24	−0.63(−0.82, −0.43)^§^	39(23.6)	0.4(0.2, 0.6)^§^
Rapid growth	252	0.61 ± 1.17	0.24(0.07, 0.41)^‡^	95(37.7)	1.6(1.1, 2.2)^‡^
Extremely rapid	788	0.91 ± 1.24	0.89(0.74, 1.04)^§^	380(48.2)	4.7(3.4, 6.3)^§^
Weight gain *z*-score			0.40(0.36, 0.45)^§^		2.0(1.8, 2.2)^§^

Gestational age category was defined as full term (gestational age ≥39 weeks), early term (37–38 weeks), late preterm (34–36 weeks), and early preterm (<34 weeks). Weight gain *z*-score was defined as the change in weight for age *z*–score between birth and 4 months and was categorized into four groups: slow (weight gain *z*-score less than −0.67); on track (weight gain *z*-score between −0.67 and 0.67), rapid (weight gain *z*-score between 0.67 and 1.28), and extremely rapid (weight gain *z*-score more than 1.28).

Adjusted for maternal education, race, smoking, parity, prepregnancy BMI category, hypertensive disorders, diabetes, fetal growth status, gestational age categories, and breastfeeding status.

^†^P < 0.05; ^‡^P < 0.01; ^§^P < 0.001.

**Table 5:** The association between weight gain in the first 4 months and leptin, adiponectin and adiponectin/leptin ratio at median (interquartile range) age: 1.7 (0.9–3.5) years.

**Weight gain Category**		**Lnleptin(n = 1136)**			** Lnadiponectin (n = 1139)**			**Ln(adiponectin/leptin ratio) (n = 1092)**								
	**N**	**Crude**	**Adjusted**	**N**	**Crude**	**Adjusted**	**N**	**Crude**	**Adjusted**							
		**β(95%CI)**	**β(95%CI)**		**β(95%CI)**	**β(95%CI)**		**β(95%CI)**	**β(95%CI)**							
Total sample*									
On track	413	ref	ref	412	ref	ref	397	ref	ref
Slow growth	239	−0.17(−0.34, 0.00)^†^	−0.22(−0.38, −0.05)^‡^	241	−0.10(−0.21, 0.01)	−0.09(−0.20, 0.02)	230	0.07(−0.15, 0.28)	0.12(−0.09, 0.33)
Rapid growth	168	0.16(−0.03, 0.34)	0.20(0.01, 0.39)^†^	170	−0.06(−0.19, 0.07)	0.00(−0.13, 0.13)	161	−0.22(−0.46, 0.03)	−0.21(−0.45, 0.03)
Extremely rapid	316	0.30(0.14, 0.45)^§^	0.36(0.19, 0.53)^§^	316	−0.07(−0.17, 0.04)	−0.02(−0.14, 0.10)	304	−0.40(−0.60, −0.20)^§^	−0.39(−0.61, −0.17)^§^
Weight gain *z*-score		0.14(0.09, 0.18)^§^	0.18(0.13, 0.23)^§^		0.00(−0.03, 0.03)	0.01(−0.02, 0.05)		−0.15(−0.20, −0.09)^§^	−0.17(−0.24, −0.10)^§^
Full term									
On track	229	ref	ref	231	ref	ref	221	ref	ref
Slow growth	169	−0.13(−0.34, 0.08)	−0.21(−0.42, 0.00)^†^	170	−0.15(−0.28, −0.01)^†^	−0.14(−0.27, 0.00)	161	−0.02(−0.28, 0.25)	0.07(−0.20, 0.33)
Rapid growth	74	0.36(0.08, 0.64)^†^	0.39(0.12, 0.66)^‡^	73	−0.08(−0.26, 0.10)	−0.06(−0.24, 0.12)	70	−0.45(−0.80, −0.10)^†^	−0.46(−0.80, −0.11)^†^
Extremely rapid	58	0.28(−0.02, 0.59)	0.25(−0.05, 0.56)	58	0.03(−0.17, 0.23)	0.00(−0.20, 0.20)	56	−0.24(−0.62, 0.15)	−0.24(−0.63, 0.14)
Weight gain *z*-score		0.13(0.06, 0.21)^§^	0.16(0.08, 0.24)^§^		0.06(0.01, 0.11)^†^	0.06(0.00, 0.11)^†^		−0.07(−0.17, 0.03)	−0.10(−0.20, 0.00)
Early term									
On track	123	ref	ref	121	ref	ref	118	ref	ref
Slow growth	55	−0.21(−0.53, 0.12)	−0.21(−0.56, 0.14)	55	−0.11(−0.36, 0.13)	0.02(−0.24, 0.28)	54	0.10(−0.33, 0.53)	0.25(−0.20, 0.71)
Rapid growth	55	0.01(−0.31, 0.34)	0.07(−0.25, 0.39)	58	−0.06(−0.30, 0.18)	−0.03(−0.27, 0.21)	53	−0.06(−0.50, 0.37)	−0.09(−0.51, 0.33)
Extremely rapid	74	0.33(0.03, 0.62)^†^	0.42(0.13, 0.72)^‡^	75	−0.04(−0.26, 0.18)	0.00(−0.22, 0.22)	73	−0.37(−0.77, 0.02)	−0.40(−0.79, −0.01)^†^
Weight gain *z*-score		0.15(0.06, 0.24)^‡^	0.19(0.09, 0.28)^§^		−0.01(−0.08, 0.06)	−0.03(−0.11, 0.04)		−0.17(−0.29, −0.05)^§^	−0.22(−0.35, −0.10)^§^
Late preterm									
On track	47	ref	ref	47	ref	ref	46	ref	ref
Slow growth	14	−0.20(−0.82, 0.42)	−0.24(−0.86, 0.39)	15	0.01(−0.38, 0.40)	0.26(−0.10, 0.63)	14	0.19(−0.58, 0.96)	0.50(−0.27, 1.26)
Rapid growth	29	−0.14(−0.62, 0.34)	−0.12(−0.62, 0.37)	29	0.01(−0.30, 0.32)	0.13(−0.17, 0.43)	28	0.14(−0.46, 0.74)	0.25(−0.36, 0.86)
Extremely rapid	84	0.37(0.00, 0.74)^†^	0.37(−0.03, 0.76)	84	−0.02(−0.26, 0.22)	−0.02(−0.25, 0.22)	82	−0.43(−0.89, 0.03)	−0.40(−0.89, 0.09)
Weight gain *z*-score		0.22(0.10, 0.33)^§^	0.23(0.11, 0.35)^§^		−0.01(−0.09, 0.06)	−0.05(−0.12, 0.03)		−0.23(−0.37, −0.09)^‡^	−0.27(−0.42, −0.12)^§^
Early preterm									
On track	14	ref	ref	13	ref	ref	12	ref	1.0
Slow growth	1	–	–	1	–	–	1	–	–
Rapid growth	10	0.19(−0.65, 1.03)	0.04(−0.82, 0.89)	10	0.27(−0.34, 0.88)	0.55(−0.10, 1.20)	10	0.06(−1.08, 1.20)	0.40(−0.80, 1.59)
Extremely rapid	100	0.18(−0.40, 0.76)	0.00(−0.59, 0.59)	99	0.09(−0.33, 0.52)	0.12(−0.34, 0.58)	93	−0.20(−1.02, 0.61)	−0.09(−0.95, 0.77)
Weight gain *z*-score		0.20(0.03, 0.37)^†^	0.16(0.00, 0.33)		−0.04(−0.16, 0.08)	−0.07(−0.20, 0.05)		−0.28(−0.51, −0.04)^†^	−0.27(−0.50, −0.05)^†^

Gestational age category was defined as full term (gestational age ≥39 weeks), early term (37–38 weeks), late preterm (34–36 weeks), and early preterm (<34 weeks). Weight gain *z*-score was defined as the change in weight for age *z*–score between birth and 4 months and was categorized into four groups: slow (weight gain *z*-score less than −0.67); on track (weight gain *z*-score between −0.67 and 0.67), rapid (weight gain *z*-score between 0.67 and 1.28), and extremely rapid (weight gain *z*-score more than 1.28).

Adjusted for maternal education, race, smoking, parity, prepregnancy BMI category, hypertensive disorders, diabetes, fetal growth, and breastfeeding status.

*Additional adjustment for gestational age categories. ^†^P < 0.05; ^‡^P < 0.01; ^§^P < 0.001.
